# A computational-first proof-of-concept for dual-function metal chelation and aggregate neutralization in an Aβ42 *Drosophila* model

**DOI:** 10.3389/fnagi.2026.1912315

**Published:** 2026-09-22

**Authors:** Edric Tsai, Zimu Wang, James Truglio

**Affiliations:** Great Neck South High School, Great Neck, NY, United States

**Keywords:** Alzheimer’s disease, amyloid-β (Aβ42), metal chelation, maltol, protoporphyrin IX, photodynamic therapy, *Drosophila melanogaster*, density functional theory (DFT)

## Abstract

Alzheimer’s disease (AD) pathology is amplified by a self-reinforcing metal–Aβ–ROS axis, in which redox-active Cu and Fe bound to Aβ N-terminal histidines catalyze Fenton-like ROS generation and accelerate aggregation. Existing single-target interventions – anti-amyloid antibodies, antioxidants, metal-protein attenuating compounds, aggregation inhibitors, and photooxygenation catalysts – each address only one node of this loop. We used a computational-first framework to evaluate a dual-function strategy targeting two connected points of the axis: upstream redox-active metal sequestration by maltol, and downstream photodynamic modification of Aβ aggregates via endogenous protoporphyrin IX (PpIX), generated from 5-aminolevulinic acid (5-ALA) under 400 nm illumination. *Drosophila* experiments served as functional validation, testing whether this combined intervention improved Aβ42-associated behavioral and survival outcomes and was tolerated in wild-type (WT) flies. DFT calculations (B3LYP-D3BJ/def2-SVP//def2-TZVP, CPCM/SMD water) showed deprotonated maltol localizes oxygen-donor electron density comparable to deprotonated deferiprone, and forms a converged, solvent-stabilized Fe(III)–maltol complex. Cu(II) and Zn(II) coordination was weak and geometrically incomplete, indicating an Fe-preferential rather than broadly nonspecific chelation profile; the study does not demonstrate protection against Cu(II)-mediated ROS. Time-dependent DFT predicted PpIX excitations spanning the visible/near-UV range consistent with photosensitizer behavior, and docking against Aβ42 fibril 5OQV showed PpIX engaged the central hydrophobic/turn surface more strongly than chlorin e6 or maltol. In a pan-neuronal Aβ42 *Drosophila* model, dual treatment improved climbing performance (*p* = 2.39 × 10^−3^ vs. untreated Aβ42) and extended lifespan (log-rank *p* = 6.04 × 10^−13^; median survival ∼40 d); WT + Treatment flies appeared tolerant, though no formal statistical contrast was pre-specified. Treatment-group ThT amyloid burden was not measured, so no amyloid-reduction claim is made. Because chelation-only and 5-ALA/light-only arms were not included, we cannot attribute improvement to either intervention alone and cannot make a formal claim of additive or interactive effects between the two intervention components. The combined intervention was associated with improved behavioral and survival outcomes in the Aβ42 *Drosophila* model. Because the present study lacks single-intervention controls and treatment-group pathological measurements, these findings do not establish the mechanism responsible for the observed functional improvement. The study illustrates how ground-state, excited-state, and docking calculations can jointly inform a computationally motivated intervention tested in a tractable organismal model.

## 1 Introduction

Alzheimer’s disease (AD) is the most common form of dementia and affects more than 57 million people worldwide, with prevalence projected to exceed 150 million by 2050 (Nichols et al., 2022; World Health Organization, 2025). Although AD is clinically heterogeneous, its molecular pathology converges on a small set of interacting features: extracellular amyloid-β (Aβ) deposition derived from amyloid precursor protein (APP), intracellular tau pathology, mitochondrial dysfunction, neuroinflammation, oxidative stress, and dyshomeostasis of redox-active metals (Scheltens et al., 2021; Karran and De Strooper, 2022). Among these, the mechanistic link with the strongest chemical grounding is the metal–Aβ–ROS axis. Aβ, particularly the more aggregation-prone Aβ42, coordinates Cu(I/II), Fe(II/III), and Zn(II) through histidine residues near the N-terminus (H6, H13, H14); redox-active Fe and Cu, when ligated to Aβ, catalyze Fenton-like chemistry that produces hydroxyl radical and other reactive oxygen species (ROS) at the aggregate surface; and the resulting oxidative damage destabilizes proteostasis and accelerates further aggregation (Huang et al., 1999; Bush, 2003; Faller et al., 2014). The loop is self-amplifying, suggesting that Aβ toxicity is not determined solely by peptide concentration but by the chemical environment, particularly the redox-active metal pool, in which Aβ assembles. Zinc contributes through a distinct mechanism: it is not redox-active under normal physiological conditions but can stabilize Aβ aggregates and modulate synaptic signaling (Bush et al., 1994; Atwood et al., 1998).

Therapeutic strategies that target a single node of this loop have produced partial benefit at best. Anti-Aβ monoclonal antibodies such as lecanemab and donanemab clear plaque and slow clinical decline modestly in selected early-symptomatic patients but do not reverse neurodegeneration and leave downstream oxidative and metal-catalyzed pathology intact (van Dyck et al., 2023; Sims et al., 2023; Karran and De Strooper, 2022). Broad-spectrum antioxidants, α-tocopherol most prominently, delay functional decline marginally in some trials but do not block upstream ROS generation, likely because ROS are short-lived and are produced locally at mitochondria, lipid membranes, inflammatory lesions, and metal-rich Aβ assemblies (Sano et al., 1997; Dysken et al., 2014). Metal-protein attenuating compounds (clioquinol, PBT2) demonstrated that pathological metal–Aβ interactions are druggable but did not establish disease modification at the clinical endpoint (Ritchie et al., 2003; Lannfelt et al., 2008). Aggregation inhibitors such as scyllo-inositol did not meet primary efficacy endpoints in phase 2 (Salloway et al., 2011). Photooxygenation catalysts can chemically modify pre-existing Aβ assemblies (Taniguchi et al., 2014; Ni et al., 2018; Nagashima et al., 2021), but delivery, light access, and selectivity remain limiting. The pattern is consistent: amyloid biology matters, redox biology matters, metal biology matters – yet intervening at any one node leaves the others active, and the self-amplifying loop persists.

Here we test whether a computationally motivated dual-function strategy could plausibly target the metal–Aβ–ROS axis at two mechanistically connected points: upstream redox-active metal sequestration by maltol, and downstream photodynamic modification of pre-existing Aβ aggregates via the porphyrin protoporphyrin IX (PpIX). Maltol is capable of coordinating transition metals through a bidentate oxygen-donor motif structurally analogous to the hydroxypyridinone core of the clinically used iron chelator deferiprone; its moderate affinity is, in principle, desirable: strong enough to plausibly influence redox-active metal chemistry but weak enough to leave essential metalloproteins largely intact. PpIX is the endogenous porphyrin product of the heme biosynthetic pathway and a well-characterized photosensitizer with a Soret band near 400 nm, visible-light Q-bands, and high singlet-oxygen quantum yield (Dougherty et al., 1998; Agostinis et al., 2011). To avoid the delivery and bioavailability problems that limit direct porphyrin administration, we drove endogenous PpIX accumulation by feeding 2 mM 5-aminolevulinic acid (5-ALA) and triggered photodynamic activity with 5-min, 400 nm illumination at ∼10–20 mW/cm^2^ (≈3–6 J/cm^2^). The computational framework predicts that one component could influence redox-active metal coordination while the second could plausibly interact with aggregate surfaces; whether either action occurs *in vivo* is not tested experimentally in this work. Because the present design lacks chelation-only and 5-ALA/light-only arms, we report a combined-treatment effect and make no formal claim of additive or interactive effects between the two intervention components.

This work is best read as a computational-first design study with functional biological validation. The computational analyses – ground-state DFT for chelation, TD-DFT for excited-state photodynamic plausibility, and molecular docking for Aβ42 surface engagement – are the primary contribution; they specify the mechanistic hypothesis, the metal-preference expectations, and the aggregate-surface expectations that motivate the combined treatment. The wet-lab component then addresses a narrower validation question: whether the computationally supported dual treatment produces a measurable functional and survival signal in an intact organism model of Aβ42 toxicity, and whether it is apparently tolerated in the absence of Aβ42 expression. It is not intended to isolate the mechanism of action of either arm; that requires single-intervention arms, treatment-group amyloid quantification, and Cu(II)- and Zn(II)-directed chemistry that the present design does not contain. Pathological (Thioflavin T (ThT) amyloid burden), behavioral (negative-geotaxis climbing over 30 d), and organismal (lifespan, *n* = 50 per group) endpoints are collected in a *Drosophila melanogaster* model expressing human Aβ42 pan-neuronally (UAS-Aβ42 responder, BDSC #33769; driver line BDSC #458; Iijima et al., 2004; Crowther et al., 2005). We hypothesized that the computationally designed combined intervention would improve behavioral and survival outcomes in the Aβ42 *Drosophila* model without obviously impairing WT phenotypes.

## 2 Materials and methods

### 2.1 Computational chelation: ligand and complex geometries

Maltol and structurally related candidate ligands were built in Avogadro and geometry-optimized in ORCA 6.1.0 (Neese, 2022). Deferiprone was included as a clinically used Fe(III) chelator reference. Geometry optimizations used the B3LYP hybrid functional with Grimme D3 dispersion and Becke–Johnson damping (D3BJ), the def2-SVP basis set, and TightSCF convergence criteria. Higher-level single-point, frequency, and solvent-corrected calculations used B3LYP-D3BJ with def2-TZVP, the RIJCOSX approximation, and TightSCF. Both gas-phase and implicit-solvent calculations were performed; solvent corrections were applied using CPCM with SMD water (ε = 78.3550). Most jobs were run on a single processor core, with selected jobs run on four cores. Optimized geometries were inspected for unrealistic bond lengths, distorted ring systems, or convergence artifacts before further analysis. The B3LYP/def2-SVP (opt) and def2-TZVP (single-point) combination is appropriate for qualitative geometric and electronic trends within a related ligand series; higher-level correlated methods (e.g., DLPNO-CCSD(T)/def2-TZVPP) would be required to obtain quantitative chelation-strength ordering for first-row transition metals and are noted here as a natural extension of this workflow.

For each optimized ligand we extracted total electronic energy, HOMO and LUMO energies, orbital surfaces, the HOMO–LUMO gap, the dipole moment, the molecular electrostatic potential, and CHELPG atomic charges (Breneman and Wiberg, 1990). CHELPG analyses were performed for maltol, deprotonated maltol, deferiprone, and deprotonated deferiprone. Donor-atom partial charges and HOMO localization were used to predict which oxygen atoms would coordinate the metal center.

Metal complexes were built by placing Fe(III), Cu(II), or Zn(II) – Fe and Cu treated as redox-active and Zn as redox-inactive – at chemically reasonable starting positions relative to the optimized ligand donor atoms, and the complexes were geometry-optimized using the same functional, basis set, and convergence workflow. Fe-containing systems were treated as high-spin, Cu(II)-containing systems as doublets, and closed-shell organic ligands or Zn(II)-containing systems as singlets unless the input structure required otherwise. Complexes that distorted or dissociated during optimization were flagged; distorted complexes were retained and reported (rather than excluded), because non-convergence to a chemically reasonable coordination geometry is itself informative about the ligand’s metal preference under this modeling setup. Binding energies were computed as


ΔE=bindE−complex(E+metaln⋅E)ligand,

where E_complex_, E_metal_, and E_ligand_ are optimized electronic energies, and n is the number of ligand units in the coordination sphere. More negative ΔE_*bind*_ values were interpreted as more favorable association under the modeled conditions and used only for relative comparison within the ligand series, not as absolute thermodynamic binding constants. Metal–donor bond lengths and coordination geometries were recorded for each converged complex. Because solvent-corrected binding energies can be unstable for isolated metal ions, especially Fe(III), solvent-corrected values were interpreted cautiously and comparatively.

### 2.2 Metal selectivity and competition with an Aβ histidine mimic

Relative metal selectivity within each ligand was estimated as


ΔΔE=selectivityΔE(metalA)bind−ΔE(metalB)bind,

with more negative values indicating preference for metal A over metal B. To test whether each ligand could plausibly compete with Aβ N-terminal metal coordination, an imidazole fragment was used as a minimal mimic of the Aβ histidines H6/H13/H14. Metal–imidazole complexes were built and optimized as the simplified Aβ-mimic species, and a metal-transfer reaction was evaluated:


metal−(imidazole)+mligand→metal−ligand+m⋅imidazole

ΔE=competition[E(metal−ligand)+m⋅E(imidazole)]

−[E(metal−(imidazole))m+E(ligand)].

A negative ΔE_*competition*_ indicates that metal transfer from the imidazole mimic to the candidate ligand is energetically favorable under the modeled conditions. Imidazole is an intentionally minimal proxy: it captures the metal-coordinating side chain of Aβ histidines but does not reproduce Aβ backbone geometry, N-terminal chelation with backbone amides, local pH, side-chain electrostatics from neighboring D1/E3/D7 residues, or aggregate-state effects. Competition energies computed against imidazole are therefore interpreted only as evidence of chemical plausibility, not as *in vivo* competition with Aβ. A short N-terminal peptide mimic such as DAEFRHDSGY (Aβ1–10) – which retains the H6-containing coordination environment together with the flanking acidic residues that shape the effective p_*K_a_*_ and electrostatics of the metal-binding pocket – would be the appropriate next-level substrate and is noted here as immediate future work. Donor–metal bond lengths, retention of coordination geometry, HOMO/LUMO localization, and relative competition energies were used together to flag ligands that bound indiscriminately across all three metals.

### 2.3 PpIX excited-state analysis

Protoporphyrin IX (PpIX) was modeled using the protonation state defined in the prepared input structure, geometry-optimized using the same DFT workflow described in section “2.1 Computational chelation: ligand and complex geometries,” and inspected for macrocycle planarity and side-chain (propionate, vinyl) conformation before excited-state analysis. Time-dependent DFT was then used to compute vertical excitation energies, oscillator strengths, and major orbital-transition assignments for the 10 lowest singlet excitations (Runge and Gross, 1984; Casida, 1995). Predicted absorption wavelengths and oscillator strengths were compared with the expected porphyrin Soret band near 400 nm and Q-bands between 500 and 650 nm. Frontier orbital surfaces were inspected to confirm that the lowest-lying excitations involve the conjugated porphyrin core, consistent with photosensitizer behavior. TD-DFT results are interpreted as evidence that the PpIX electronic structure is consistent with photoactivation. TD-DFT does not directly quantify singlet-oxygen yield, intersystem-crossing efficiency, or biological phototoxicity; those endpoints require dedicated photophysical and cellular assays.

### 2.4 PpIX–Aβ aggregate docking

Molecular docking was used to evaluate whether PpIX could plausibly localize to the surface of Aβ42 aggregates before photodynamic activation, since aggregate-association is required for light-activated chemistry to act on the aggregate. The Aβ42 aggregate receptor was prepared from the experimentally determined Aβ42 fibril structure PDB 5OQV; crystallographic waters, ions, and unrelated co-crystallized ligands were removed; missing heavy atoms were added; and hydrogens were assigned at pH 7.4. PpIX was prepared in the same protonation state used for the TD-DFT analysis (section “2.3 PpIX excited-state analysis”), energy-minimized prior to docking, and saved as a flexible ligand.

Docking was performed in PyRx using AutoDock Vina (Trott and Olson, 2010). The Aβ42 aggregate was treated as the receptor and PpIX as the ligand. Aβ42 fibrils do not present a classical enzyme-like binding pocket; the search region was therefore placed over solvent-exposed β-sheet surfaces and hydrophobic grooves rather than a predefined active site, using three 25 × 25 × 25 Å search boxes centered on the histidine cluster, the KLVFFA aggregation core, and the C-terminal hydrophobic region, with exhaustiveness 8 and the top 9 poses retained per run. The lowest-energy and most structurally plausible poses were visualized in BIOVIA Discovery Studio Visualizer to identify hydrophobic, π–π, hydrogen-bonding, and electrostatic contacts. Because AutoDock Vina scoring is calibrated on well-defined ligand–pocket binding events, docking scores against a flat, pocket-less aggregate surface should be interpreted qualitatively – that is, as evidence for relative surface localization and residue-level contact patterns across ligands, not as quantitative affinity values. A pose was considered plausible only if PpIX remained in direct surface contact with the aggregate.

### 2.5 *Drosophila* genetics, husbandry, and treatment groups

The Aβ42 responder line UAS-Aβ42 (Bloomington *Drosophila* Stock Center #33769) and the pan-neuronal GAL4 driver line BDSC #458 were obtained from the Bloomington *Drosophila* Stock Center and crossed to generate F1 progeny expressing human Aβ42 pan-neuronally (Brand and Perrimon, 1993; Iijima et al., 2004; Crowther et al., 2005). Both male and female flies were used in proportions consistent with those in the F1 progeny; sex-stratified analyses were not pre-specified for the primary endpoints. Flies were collected within 24 h of eclosion, placed into treatment or control vials, and maintained at room temperature under a 9 h light/15 h dark cycle and consistent laboratory conditions, with all groups housed in the same room.

Standard fly food medium was prepared by boiling distilled water and adding agar with constant stirring until fully dissolved. Brewer’s yeast and sucrose were then added, and the mixture was stirred until it returned to a boil. The medium was cooked with continued stirring for approximately 15 min. Tegosept (p-hydroxybenzoic acid methyl ester) was dissolved in ethanol and added to the medium as an antimicrobial, followed by thorough mixing. The prepared medium was dispensed into standard culture vials and capped. Treatment compounds were incorporated into the food medium so that flies were continuously exposed from eclosion.

Four primary groups were established: (i) WT controls; (ii) WT receiving the dual treatment (treatment-tolerance control); (iii) untreated Aβ42 disease-model flies; and (iv) Aβ42 flies receiving the dual treatment. The chelation arm consisted of 10 μM EDTA and 10 mM maltol incorporated into the food. EDTA is a broad-spectrum chelator administered here at a low dietary concentration; this arm is therefore treated operationally as a “chelation environment,” not as evidence for selective *in vivo* maltol action. The photodynamic arm consisted of 2 mM 5-ALA in the food to drive endogenous PpIX accumulation, followed by 400 nm illumination for 5 min at ∼10–20 mW/cm^2^ (∼3–6 J/cm^2^ estimated fluence) once daily during the treatment period. Dual-treatment flies received both the chelation food and the 5-ALA/light protocol. Chelation-only and 5-ALA/light-only Aβ42 arms are not present in the current design; the combined-treatment effect is therefore reported without a formal claim of additive or interactive effects between the two intervention components, and mechanistic attribution to either single intervention is not attempted (see section “2.9 Statistical analysis”).

### 2.6 Thioflavin T fluorescence-based amyloid burden

Amyloid burden was estimated using a Thioflavin T (ThT) fluorescence assay; ThT binds β-sheet-rich amyloid structures and fluoresces upon association with aggregates. Flies were collected after death from the climbing and lifespan vials and stored at room temperature until processing. For each group, flies were segregated by behavioral trial and pooled, generating three trial-level biological replicates per group; each pool represents one independent vial replicate rather than an individual-fly measurement. Brain counts were recorded per pool and used for normalization.

Pooled samples were homogenized in approximately 2.5 mL of PBS at pH 7.4 by manual mechanical homogenization. ThT was added to each homogenate at a final concentration of 10 μM, and all groups were incubated for 5 min at room temperature. Fluorescence was measured on a Vernier Go Direct SpectroVis Plus spectrophotometer (model GDX-SVISPL), with emission recorded at 482 nm and wavelength smoothing set to 8. Buffer + ThT blanks without a biological sample were included and subtracted as background. Amyloid burden was normalized to brain count per pool:

Normalized ThT amyloid burden = (background-corrected ThT fluorescence)/(number of brains).

In the present study, ThT was measured in WT and untreated Aβ42 flies as a disease-model validation endpoint and was not measured in the treatment groups. Consequently, ThT is used only to confirm that the Aβ42 line generates β-sheet-rich aggregate load above WT baseline; it is not used to evaluate treatment-mediated aggregate reduction. Because the assay reads bulk homogenate fluorescence rather than imaging single aggregates, the value is a biochemical estimate of β-sheet-rich aggregate load; it does not provide spatial localization or discriminate plaque-like assemblies from smaller fibrillar species.

### 2.7 Negative-geotaxis climbing assay

Climbing performance was measured daily for 30 d post-eclosion in WT, WT + Treatment, untreated Aβ42, and Aβ42 + Treatment flies. For each measurement, the vial was tapped firmly to bring all flies to the bottom, and the number of flies above a 5 cm threshold was counted exactly 30 s after tapping. Each group was tested in three independent trials per day, using the same vial geometry, threshold line, tapping force, observer, and time limit. The climbing index was calculated for each trial as

Climbing index = (flies above 5 cm at 30 s)/(total flies in the vial),

and the daily group value was reported as mean ± SEM across the three trials. The primary behavioral endpoint was the area under the 30-day climbing curve (Climbing AUC = Σ daily climbing index, days 1–30), which captures the full functional trajectory rather than any single day. The slope of a linear regression of climbing index on day was reported as a decline-rate measure (less-negative slope = slower decline), and the final-day climbing index was reported as a secondary endpoint. Treatment-associated rescue was interpreted as stronger if Aβ42 + Treatment flies showed consistently higher climbing than untreated Aβ42 across the time course rather than only at one isolated time point.

### 2.8 Lifespan assay

Survival was monitored daily in 50 flies per group from eclosion until death, with treatment exposure maintained continuously through the food medium and the once-daily light schedule defined in section “2.5 *Drosophila* genetics, husbandry, and treatment groups.” Flies were counted as dead when they failed to respond to gentle disturbance of the vial; flies that escaped, were crushed during transfer, or died from clearly non-disease causes were treated as right-censored. Survival data were organized by group and individual fly identification number. Kaplan–Meier survival curves were generated for each group, and survival distributions were compared by the log-rank test. Median, mean, and 25th/75th percentile lifespans were reported as descriptive statistics. Because both sexes were included and sex was not separated in the primary survival analysis, lifespan is interpreted as a group-level organismal outcome rather than a sex-specific endpoint.

### 2.9 Statistical analysis

All analyses were performed in Python and Microsoft Excel, with α = 0.05 used as the significance threshold. For each continuous endpoint – ThT-derived amyloid burden, climbing AUC, final-day climbing index, and mean lifespan – group differences were tested by one-way ANOVA when Shapiro–Wilk normality and Levene’s equal-variance assumptions were met, and by Welch ANOVA otherwise. Post-hoc pairwise comparisons used Tukey’s HSD or Games–Howell, respectively. Survival was compared by log-rank test on the Kaplan–Meier curves generated in section “2.8 Lifespan assay.” Two contrasts were pre-specified: (1) WT versus untreated Aβ42, to confirm disease-associated decline, and (2) untreated Aβ42 versus Aβ42 + Treatment, to evaluate treatment-associated rescue. Additional pairwise tests were corrected for multiple comparisons within the post-hoc family. A WT versus WT + Treatment contrast was not pre-specified; tolerability in WT is therefore reported descriptively (section “3.6 WT + Treatment groups show apparent tolerability under descriptive comparison”).

Beyond *p*-values, biological magnitude was reported using two complementary metrics. Cohen’s d was calculated for primary two-group continuous comparisons:

Cohen’s *d* = (mean group 1 − mean group 2)/pooled SD,

and percent rescue was calculated relative to the WT baseline. For endpoints in which higher values indicate improvement (climbing, lifespan):

% rescue = ((Aβ42 + Treatment) − Aβ42)/(WT − Aβ42) × 100%.

Because treatment-group amyloid-burden measurements were not available, percent amyloid rescue and the pre-specified multi-scale rescue index that requires amyloid–behavior–lifespan correlation were not computed, and no claim of treatment-mediated amyloid reduction is made. Amyloid data are used only to validate the disease-model phenotype (section “3.5 Wet-lab functional validation: partial climbing and lifespan rescue in Ab42-expressing flies”).

The design does not include chelation-only or 5-ALA/light-only Aβ42 arms. The Aβ42 + Treatment condition is therefore interpreted as a combined-treatment effect only. No formal claim of additive or interactive effects between the two intervention components is made, and mechanistic attribution to either the chelation arm or the photodynamic arm is deferred to follow-up work with the missing single-intervention arms.

## 3 Results

### 3.1 PpIX localizes to aggregation-associated Aβ42 surfaces more strongly than chlorin e6 or maltol in qualitative docking

We first asked whether the dual-function components could plausibly localize to disease-relevant surfaces of the Aβ42 fibril. Molecular docking against PDB 5OQV was performed at three search regions: the histidine cluster associated with metal coordination, the KLVFFA aggregation core, and the hydrophobic C-terminal region. Because Aβ42 fibrils lack a classical binding pocket, docking scores are interpreted qualitatively – as relative surface-localization tendencies among the tested ligands – rather than as quantitative binding affinities.

Protoporphyrin IX produced the strongest predicted binding across all three regions (−7.961, −7.912, and −7.897 kcal/mol at the C-terminal, histidine, and KLVFFA regions, respectively; Figure 1 and Table 1). Chlorin e6, included as a literature-supported photoactive Aβ modulator (Leshem et al., 2019), was intermediate (−6.974, −6.952, and −6.978 kcal/mol). Maltol was weakest (−5.384, −5.325, and −5.276 kcal/mol). At residue level, the top-ranked PpIX poses converged on a hydrophobic, turn-associated surface – repeatedly contacting Leu17, Val18, Phe19, Phe20, Ala21, and downstream residues Asn27–Ile31 (Figure 2) – rather than remaining strictly confined to the histidine search box. Chlorin e6 showed a similar but energetically weaker contact pattern, and maltol contacted overlapping turn-region residues with binding energies consistent with limited rather than amyloid-directed surface association.

**FIGURE 1:**
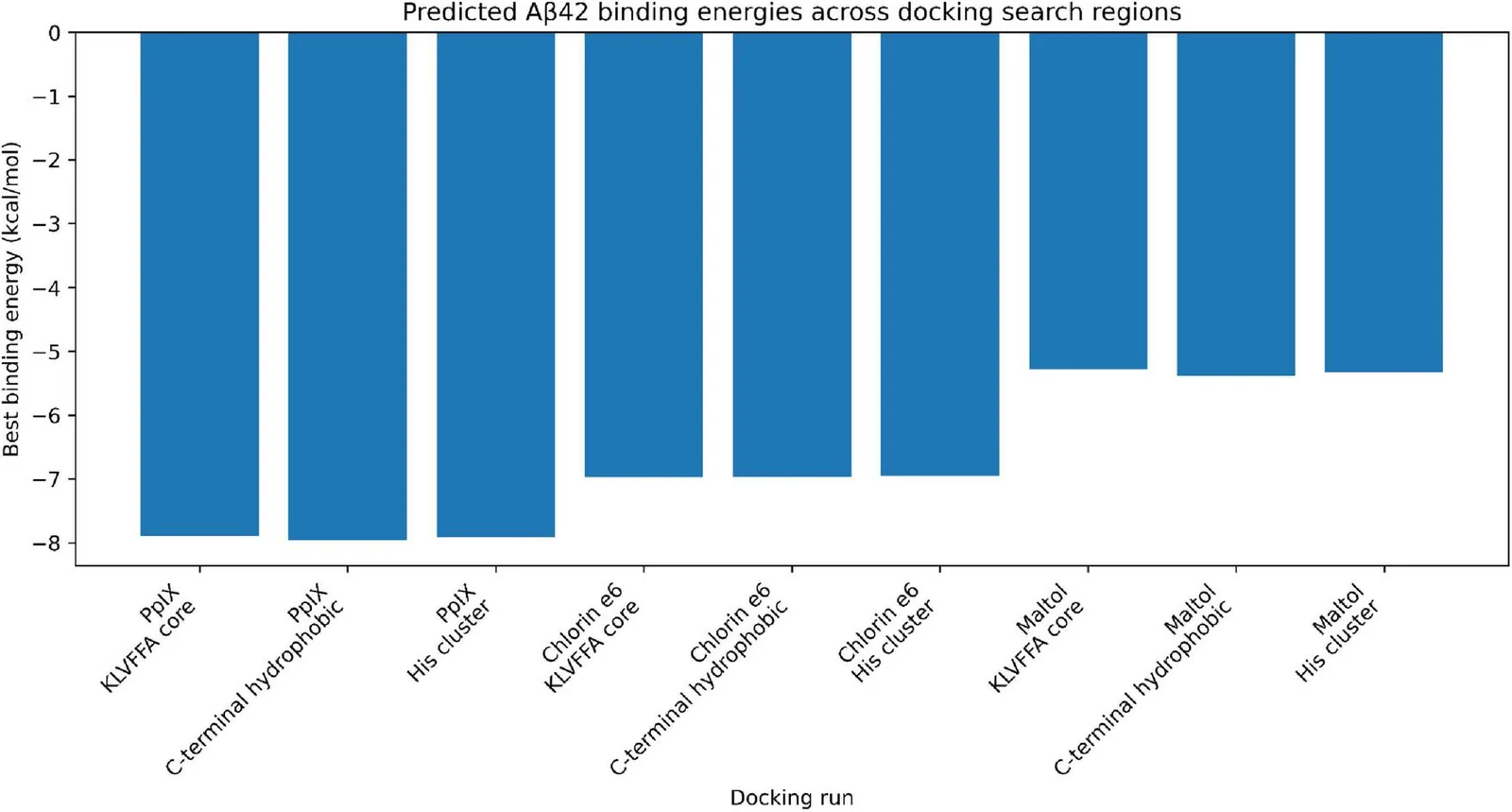
Docking-predicted Aβ42 binding energies of PpIX, chlorin e6, and maltol across the three docking search regions. Docking against Aβ42 fibril 5OQV used search regions at the histidine cluster, the KLVFFA aggregation core, and the C-terminal hydrophobic surface. More negative values indicate stronger predicted surface localization; scores are qualitative because the fibril lacks a defined pocket.

**TABLE 1:** Molecular docking scores and representative Aβ42 contact residues for PpIX, chlorin e6, and maltol against Aβ42 fibril 5OQV.

Ligand	Search region	Best binding energy (kcal/mol)	Contact residues within 4 Å
PpIX	C-terminal hydrophobic	−7.961	L17, V18, F19, F20, A21, E22, V24, N27, K28, G29, A30, I31
PpIX	His cluster	−7.912	L17, V18, F19, F20, A21, E22, V24, N27, K28, G29, A30, I31
PpIX	KLVFFA core	−7.897	L17, V18, F19, F20, A21, V24, S26, N27, K28, G29, A30, I31
Chlorin e6	C-terminal hydrophobic	−6.974	F19, A21, N27, K28, G29, A30, I31, A42
Chlorin e6	His cluster	−6.952	F19, A21, V24, S26, N27, K28, G29, A30, I31, A42
Chlorin e6	KLVFFA core	−6.978	F19, A21, N27, K28, G29, A30, I31, A42
Maltol	C-terminal hydrophobic	−5.384	F19, N27, K28, G29, A30, I31
Maltol	His cluster	−5.325	F19, N27, K28, G29, A30, I31
Maltol	KLVFFA core	−5.276	F19, N27, K28, G29, A30, I31

Because Aβ fibrils lack a classical binding pocket, docking scores are reported qualitatively and are used only for cross-ligand comparison and residue-level contact assignment. Contact residues are within 4.0 Å of the top-ranked pose.

**FIGURE 2:**
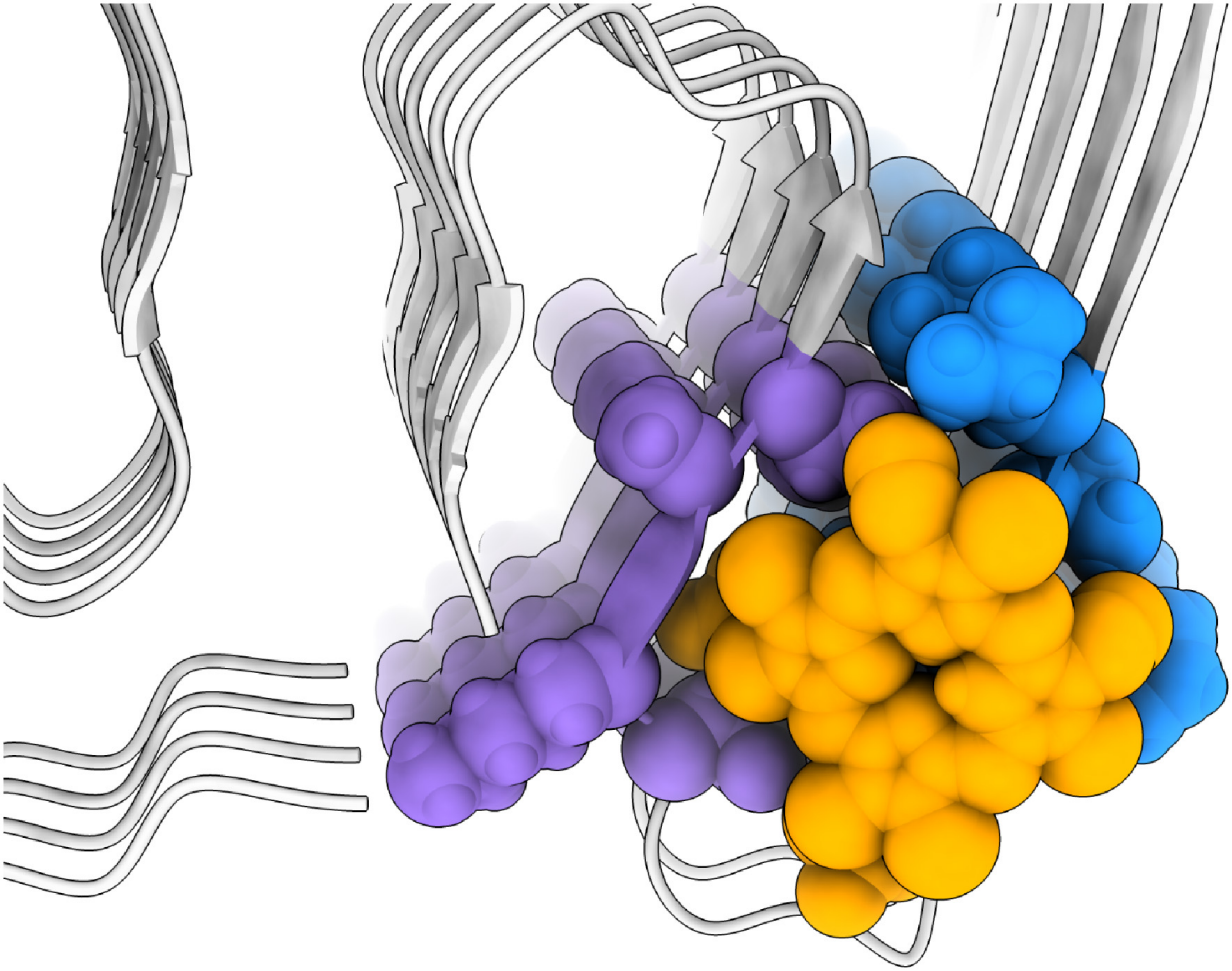
Docking-predicted top-ranked PpIX pose on the Aβ42 fibril (5OQV). PpIX localized along an aggregation-associated surface near KLVFFA-region residues Leu17–Ala21 and downstream residues Asn27–Ile31.

These docking results predict that PpIX may preferentially localize to aggregation-associated surfaces, whereas maltol is predicted to contribute primarily through metal coordination under the computational models used here (sections “3.2 Deprotonation enhances oxygen-centered donor charge in maltol to a magnitude comparable to deferiprone,” “3.3 Maltol shows Fe-preferential coordination and does not demonstrate Cu(II) or Zn(II) sequestration in the modeled complexes”). These predictions were not directly tested experimentally.

### 3.2 Deprotonation enhances oxygen-centered donor charge in maltol to a magnitude comparable to deferiprone

To examine maltol’s electronic suitability for metal coordination, we calculated CHELPG atomic charges for neutral and deprotonated maltol and compared them with deferiprone. In neutral maltol, donor-region oxygens carried CHELPG charges of −0.258, −0.462, and −0.579. Deprotonation increased oxygen-centered electron density at the two principal donor sites to −0.627 and −0.641 (Figures 3, 4 and Table 2). Deferiprone followed the same pattern: neutral donor oxygens at −0.504 and −0.554, deprotonated at −0.636 and −0.651. The magnitude of donor-site charge in deprotonated maltol is therefore comparable to that of deprotonated deferiprone, despite the smaller maltol scaffold. This pattern is consistent with oxygen-mediated metal-binding capacity and supports maltol’s proposed role as a chelating scaffold in the ground state; on its own it does not establish selectivity, which is addressed in section “3.3 Maltol shows Fe-preferential coordination and does not demonstrate Cu(II) or Zn(II) sequestration in the modeled complexes.”

**FIGURE 3:**
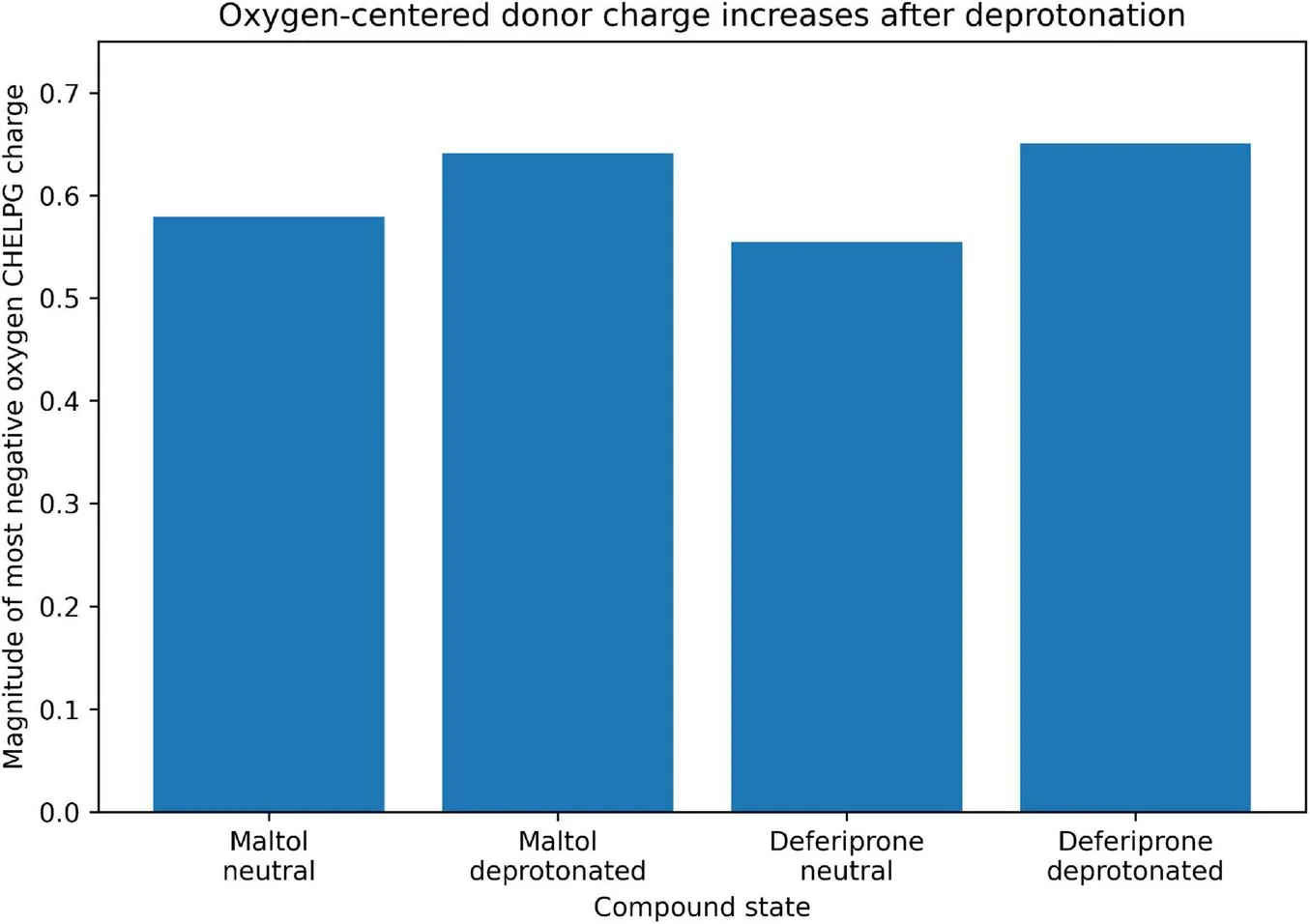
DFT-derived absolute magnitude of the most negative oxygen-donor CHELPG charge for neutral and deprotonated maltol and deferiprone. Deprotonation increased donor-site charge magnitude in both chelators.

**FIGURE 4:**
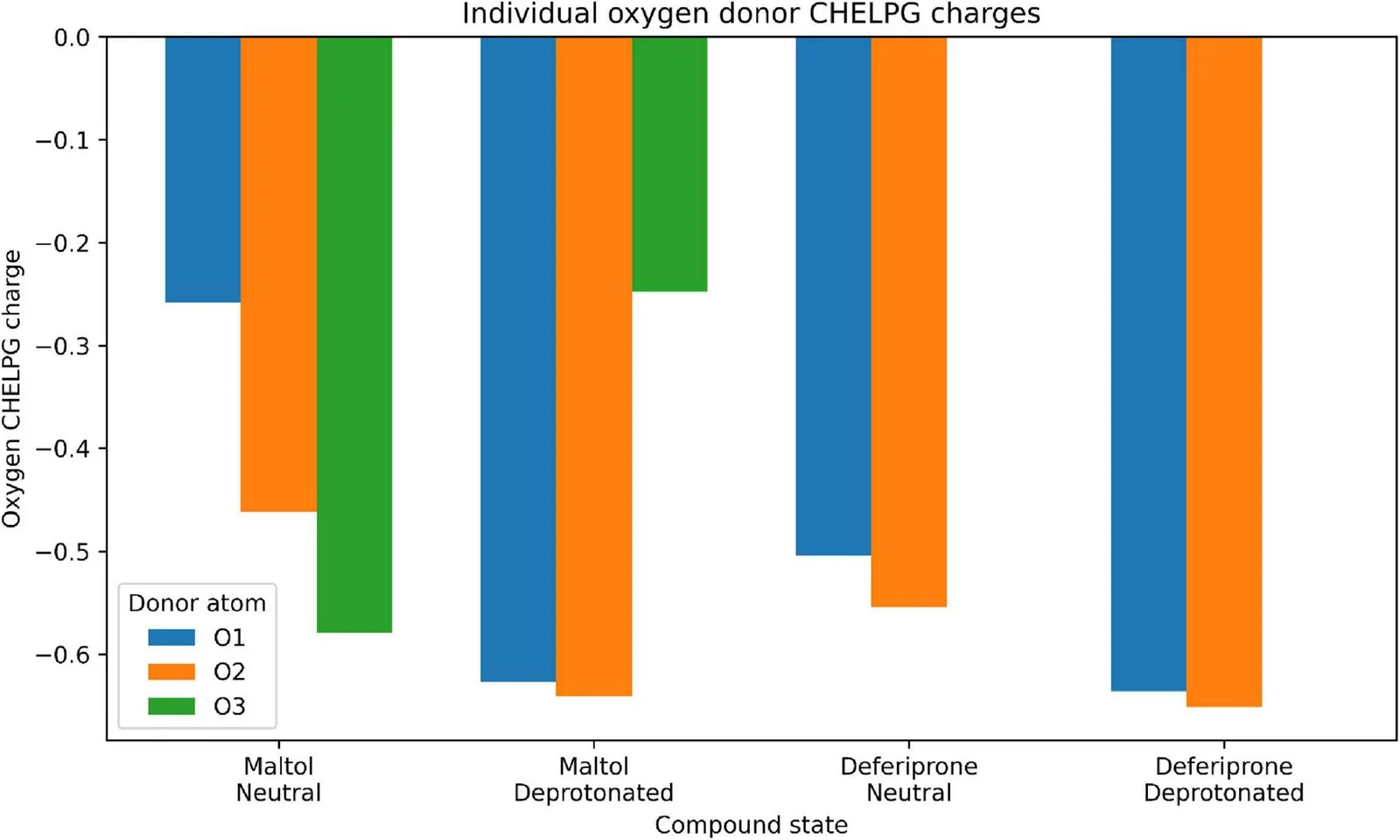
DFT-derived individual oxygen-donor CHELPG charges for neutral and deprotonated maltol and deferiprone. Deprotonated maltol shows two donor sites comparable in magnitude to deprotonated deferiprone.

**TABLE 2:** CHELPG oxygen-donor charges for neutral and deprotonated maltol compared with deferiprone.

Compound	Protonation state	Key oxygen CHELPG charges
Maltol	Neutral	−0.258, −0.462, −0.579
Maltol	Deprotonated	−0.627, −0.641, −0.248
Deferiprone	Neutral	−0.504, −0.554
Deferiprone	Deprotonated	−0.636, −0.651

More negative values indicate greater localized electron density at the donor site.

### 3.3 Maltol shows Fe-preferential coordination and does not demonstrate Cu(II) or Zn(II) sequestration in the modeled complexes

To test whether donor-site charge translated into stable metal-bound complexes, we built Fe(III)–maltol and Fe(III)–deferiprone complexes and optimized them under the workflow described in section “2.1 Computational chelation: ligand and complex geometries.” The Fe(III)–maltol complex converged to a gas-phase optimized energy of −2633.9026 E_*h*_ and a water-solvated single-point energy of −2635.6423 E_*h*_ (CPCM/SMD, ε = 78.3550), consistent with solvent stabilization of the metal complex. The Fe(III)–deferiprone reference converged at −2692.1919 E_*h*_ in gas phase and −2694.0034 E_*h*_ on the water-solvated single point (Table 3). Because Fe(III)–maltol and Fe(III)–deferiprone differ in ligand identity and molecular composition, the absolute energies are not directly comparable as a chelation-strength ranking; they support only that both ligands form converged, solvent-stabilized Fe(III) complexes. Together with the CHELPG result in section “3.2 Deprotonation enhances oxygen-centered donor charge in maltol to a magnitude comparable to deferiprone,” this provides structural and energetic plausibility for maltol’s upstream Fe-chelation role.

**TABLE 3:** Final electronic energies for the Fe(III)–maltol and Fe(III)–deferiprone complex calculations.

Complex	Calculation type	Final electronic energy (E_h)
Fe(III)–maltol	Gas-phase geometry optimization	−2633.9026
Fe(III)–maltol	Water-solvated single point (CPCM/SMD)	−2635.6423
Fe(III)–deferiprone	Gas-phase geometry optimization	−2692.1919
Fe(III)–deferiprone	Water-solvated single point (CPCM/SMD)	−2694.0034

Because ligand composition differs, absolute energies are not interpreted as direct chelation-strength rankings; both ligands form converged, solvent-stabilized Fe(III) complexes.

Cu(II) and Zn(II) complex models were then evaluated to test whether maltol showed broad nonspecific coordination of biologically relevant transition metals. Deferiprone formed well-converged Cu(II) and Zn(II) complexes with chemically reasonable metal–oxygen distances of approximately 1.94–1.97 Å for Cu–O and 1.97–2.01 Å for Zn–O. The imidazole reference complexes also showed consistent coordination geometries, with Cu–N distances near 2.01 Å, Zn–N distances near 2.02 Å, and Fe–N distances near 2.14–2.15 Å. In contrast, the optimized Cu(II)– and Zn(II)–maltol structures showed elongated and incomplete metal–oxygen coordination distances, including Cu–O distances of 2.824 and 2.999 Å and Zn–O distances of 2.970 and 2.125 Å. These distances fall well outside typical coordinate-bond ranges for Cu(II)–O and Zn(II)–O in hydroxypyridinone- and hydroxypyranone-like complexes. Cu/Zn–maltol binding and imidazole-competition energies were therefore interpreted cautiously and are not used to claim strong Cu(II) or Zn(II) sequestration by maltol. Instead, these calculations indicate that maltol supports Fe(III) complexation but does not form well-defined Cu(II) or Zn(II) complexes under this modeling setup (Figure 5). This study therefore does not provide evidence that maltol protects Aβ from Cu(II)-mediated ROS generation, and any such protection would require a distinct Cu(II)-coordinating ligand (Figure 5).

**FIGURE 5:**
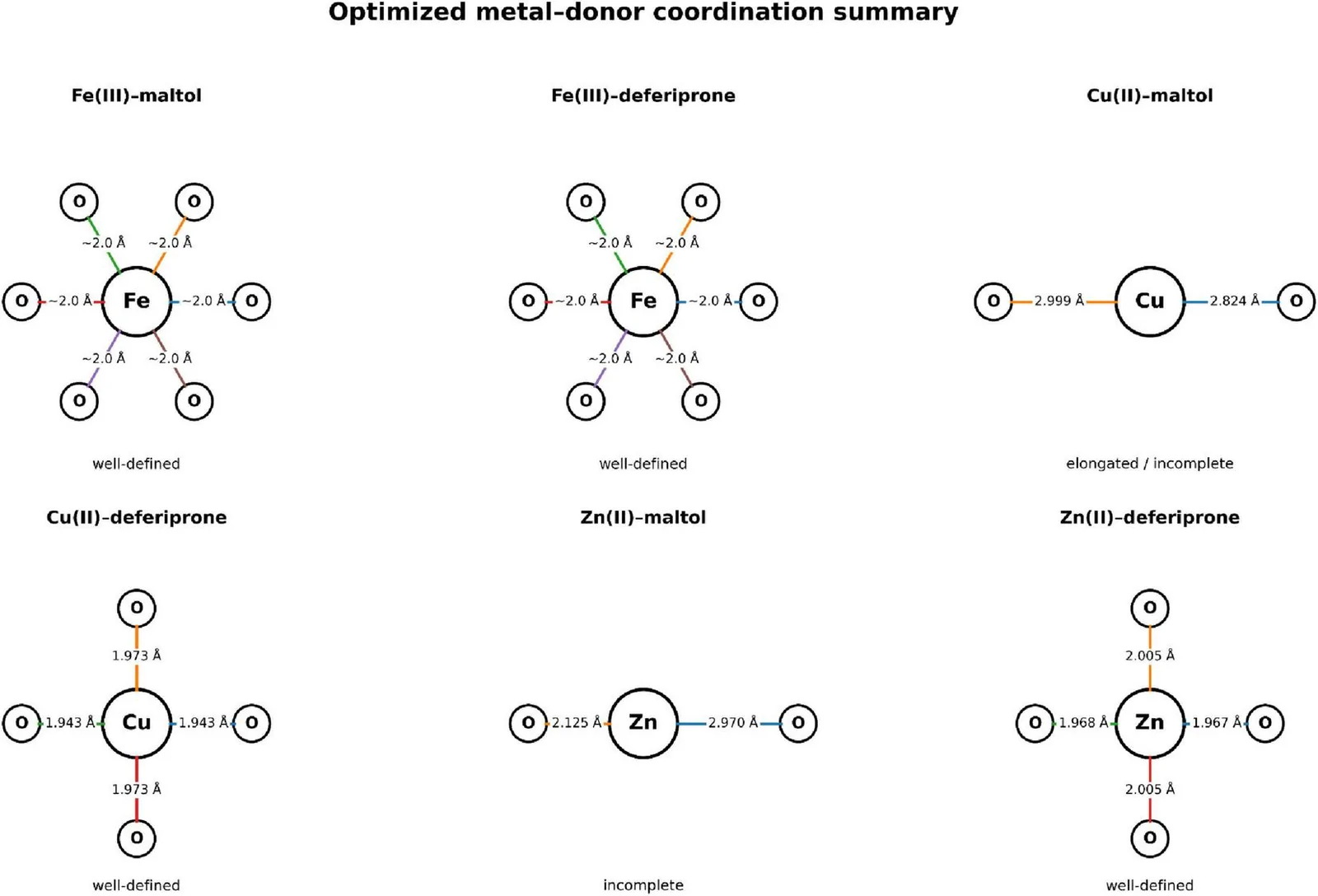
DFT-optimized coordination geometries for Fe(III)–, Cu(II)–, and Zn(II)–maltol complexes alongside the corresponding deferiprone reference complexes. Metal–donor bond lengths are labeled. Fe(III)–maltol and Fe(III)–deferiprone form well-defined complexes with typical Fe–O distances; deferiprone additionally forms Cu(II)– and Zn(II)–O complexes with distances of ∼1.94–1.97 Å (Cu–O) and ∼1.97–2.01 Å (Zn–O). In contrast, Cu(II)– and Zn(II)–maltol complexes show elongated, incomplete metal–oxygen contacts (Cu–O 2.824, 2.999 Å; Zn–O 2.970, 2.125 Å), consistent with an Fe-preferential rather than broadly nonspecific maltol chelation profile at this level of theory.

### 3.4 TD-DFT predicts visible-light absorption consistent with PpIX photosensitizer behavior

To evaluate PpIX as the photoactive component, we computed the 10 lowest singlet excitations using TD-DFT (section “2.3 PpIX excited-state analysis”). PpIX exhibited transitions spanning the visible and near-UV. The lowest-energy transitions were predicted at 519.6 nm (2.386 eV) and 496.9 nm (2.495 eV), consistent with the Q-band region of a free-base porphyrin and showing weak oscillator strengths of 0.0097 and 0.0033, respectively. Progressively stronger transitions appeared at shorter wavelengths: 391.0 nm (*f* = 0.1399), 379.2 nm (*f* = 0.2724), 361.3 nm (*f* = 0.2980), and 343.6 nm (*f* = 0.3526), with the strongest oscillator strength at 343.6 nm (Figure 6 and Table 4). This Soret-shoulder plus Q-band absorption pattern is consistent with an extended conjugated porphyrin macrocycle and supports the interpretation of PpIX as a photoactivatable species under 400 nm illumination, which lies near the Soret region.

**FIGURE 6:**
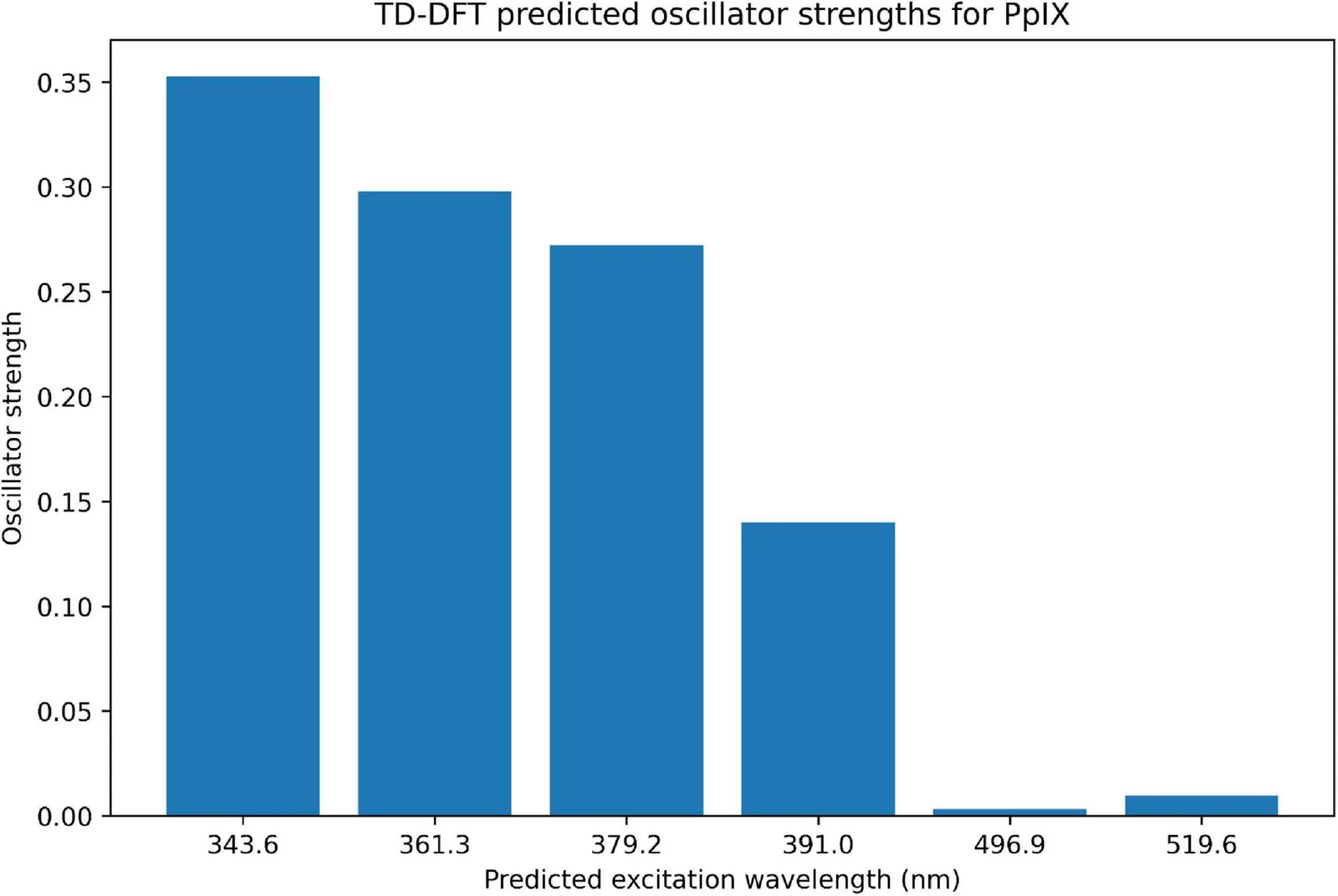
TD-DFT-predicted oscillator strengths for PpIX electronic transitions plotted against excitation wavelength. PpIX shows multiple transitions across the visible and near-UV, with stronger predicted transitions at shorter wavelengths.

**TABLE 4:** TD-DFT-predicted electronic transitions of PpIX (10 lowest singlet excitations, selected transitions shown).

Wavelength (nm)	Excitation energy (eV)	Oscillator strength (f)	Spectral region
519.6	2.386	0.0097	Q-band
496.9	2.495	0.0033	Q-band
391.0	3.171	0.1399	Soret region
379.2	3.269	0.2724	Near-UV / Soret edge
361.3	3.432	0.2980	Near-UV
343.6	3.609	0.3526	Near-UV

Higher oscillator strength indicates a more strongly allowed transition. The lowest-energy transitions at 519.6 and 496.9 nm correspond to weak Q-band excitations; the higher-energy transitions show progressively greater oscillator strengths in the Soret and near-UV regions.

Together with the docking results (section “3.1 PpIX localizes to aggregation-associated Ab42 surfaces more strongly than chlorin e6 or maltol in qualitative docking”), the TD-DFT analysis is consistent with PpIX presenting both the surface-localization profile and the excited-state structure expected of an aggregate-directed photodynamic agent. TD-DFT does not by itself establish singlet-oxygen production, intersystem-crossing efficiency, or biological phototoxicity; those endpoints require experimental confirmation.

### 3.5 Wet-lab functional validation: partial climbing and lifespan rescue in Aβ42-expressing flies

We next asked whether the chelation + 5-ALA/light treatment combination shifted Aβ42-associated functional decline *in vivo*. Untreated Aβ42 flies showed lower baseline climbing performance than WT controls and declined progressively over the 30-day assay (Figure 7). Aβ42 flies receiving the dual treatment maintained higher daily climbing indices than untreated Aβ42 flies across the time course, although they did not reach the WT trajectory; the difference between untreated and treated Aβ42 climbing trajectories was significant (*p* = 2.39 × 10^–3^).

**FIGURE 7:**
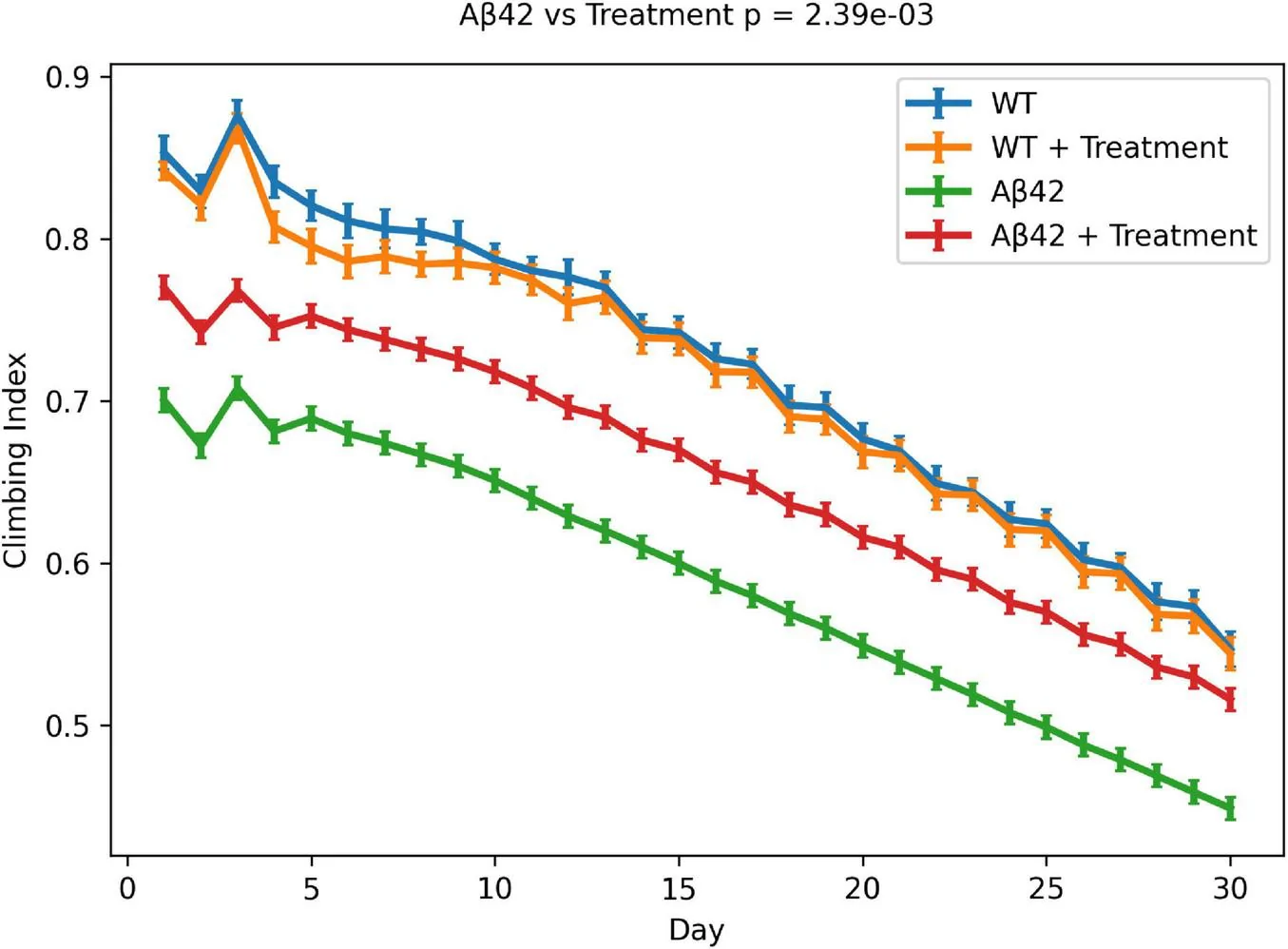
Observed climbing performance in wild-type and Aβ42-expressing flies with and without the combined intervention. Climbing index was measured daily for 30 d in WT, WT + Treatment, Aβ42, and Aβ42 + Treatment flies. Aβ42 + Treatment flies climbed higher than untreated Aβ42 flies across the time course but did not reach WT performance; no attribution of this difference to metal chelation, photodynamic action, or their interaction is made. Error bars: SEM. Displayed *p*-value corresponds to the pre-specified Aβ42 vs. Aβ42 + Treatment contrast (*p* = 2.39 × 10^−3^).

Survival followed a parallel pattern (Figure 8). Untreated Aβ42 flies showed accelerated mortality, with the Kaplan–Meier curve reaching zero substantially earlier than the WT curve. Dual treatment shifted the Aβ42 survival curve to the right without fully restoring it to the WT distribution. In the Aβ42 + Treatment group, survival remained above 50% through day 39 and crossed below 50% between days 39 and 40, giving an approximate median survival of 40 d; by day 44 the recorded survival probability was 0.018. The survival difference between untreated and treated Aβ42 flies was highly significant by log-rank test (*p* = 6.04 × 10^–13^).

**FIGURE 8:**
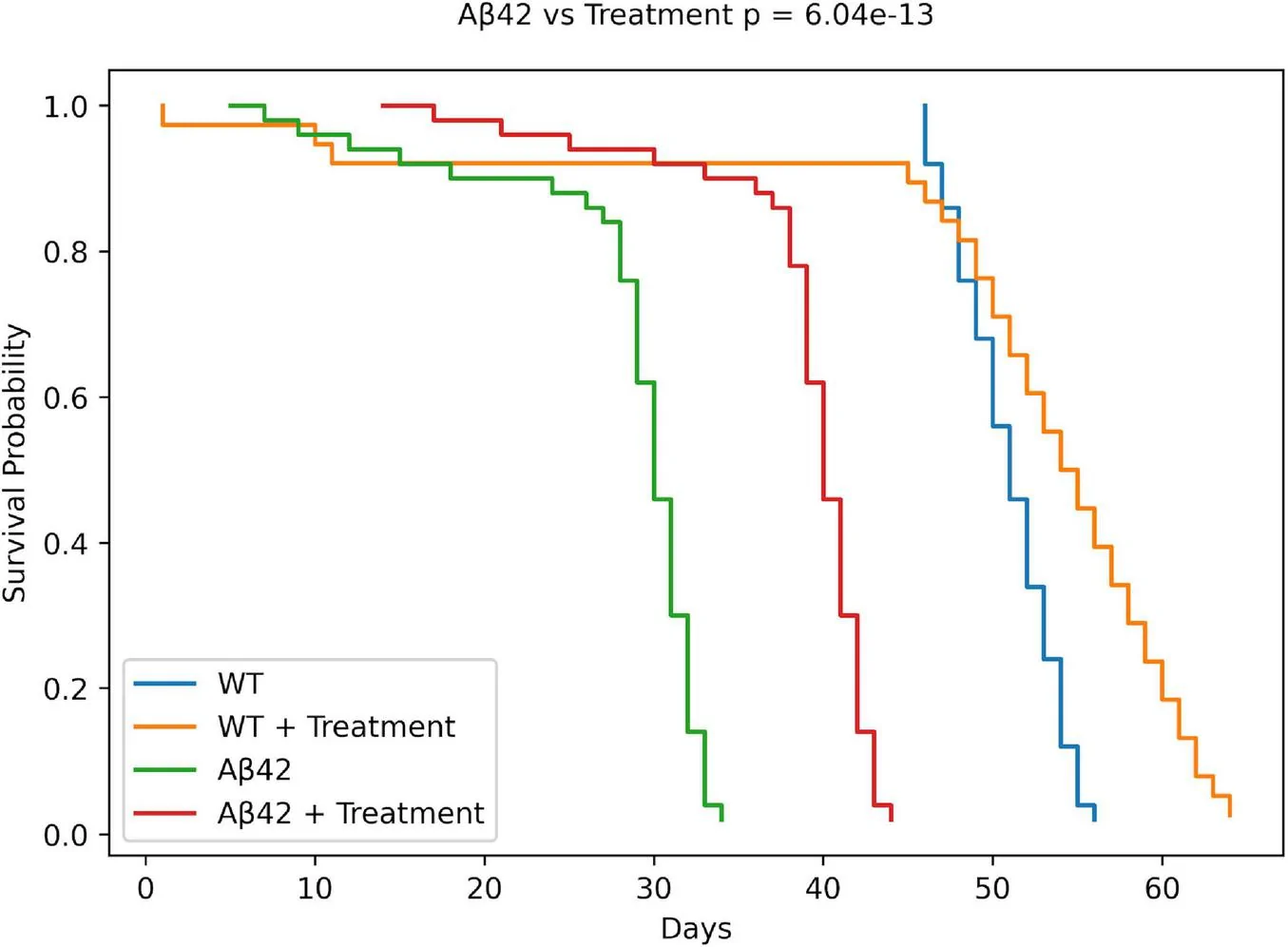
Observed Kaplan–Meier survival curves for WT, WT + Treatment, Aβ42, and Aβ42 + Treatment flies. The Aβ42 + Treatment curve is shifted rightward relative to the untreated Aβ42 curve without fully overlapping the WT distribution; no attribution of this difference to metal chelation, photodynamic action, or their interaction is made. Displayed *p*-value: pre-specified Aβ42 vs. Aβ42 + Treatment contrast (log-rank, *p* = 6.04 × 10^−13^).

Both endpoints are consistent with a partial-rescue phenotype: the dual treatment was associated with reduced – but not eliminated – functional consequences of Aβ42 expression. In the Aβ42 + Treatment group, climbing performance declined from a mean climbing index of 0.769 on day 1 to 0.517 on day 30, a 32.8% decrease across the assay period. Although locomotor function still declined with age, treated Aβ42 flies retained measurable climbing ability throughout the 30-day observation window. ThT-derived amyloid burden confirmed the disease-model phenotype, with untreated Aβ42 flies showing significantly greater amyloid-associated fluorescence than WT controls (*p* = 1.99 × 10^–20^). Because treatment-group ThT was not measured, this ThT contrast is reported strictly as disease-model validation and does not support a treatment-mediated reduction in amyloid burden; percent amyloid rescue, the pre-specified multi-scale rescue index, and cross-endpoint amyloid–behavior–lifespan correlations are consequently not reported. The behavioral and survival differences are therefore descriptive of a functional and survival improvement in the treated Aβ42 group; no mechanistic attribution to metal chelation, photodynamic action, or their interaction is made.

### 3.6 WT + Treatment groups show apparent tolerability under descriptive comparison

To control for non-disease toxicity of the treatment regimen, WT and WT + Treatment groups were compared descriptively. Climbing trajectories overlapped throughout the 30-day assay, with comparable baseline indices and parallel age-associated decline (Figure 7). The WT + Treatment survival curve tracked the untreated WT curve through the early and middle portions of the assay and extended marginally beyond it at later time points (Figure 8).

These data are consistent with apparent tolerability of the combined chelation and 5-ALA/light treatment in the absence of Aβ42 expression. A formal WT vs. WT + Treatment statistical contrast was not pre-specified (section “2.9 Statistical analysis”); the WT tolerability observation is therefore reported strictly descriptively and does not license a claim of non-toxicity. Any apparent WT lifespan extension should be interpreted cautiously and not as evidence of treatment-induced lifespan benefit. Formal tolerability assessment will require pre-specified WT vs. WT + Treatment log-rank and behavioral contrasts.

## 4 Discussion

We used a computational-first design framework to specify a two-node intervention against the Aβ42-associated metal–Aβ–ROS axis, then asked whether the same combined intervention produced a functional and survival signal in a *Drosophila* Aβ42 model. Three findings survive close scrutiny. First, the ground-state and excited-state calculations partition the two ligands into non-overlapping mechanistic niches: maltol shows charge and geometry features compatible with Fe(III) coordination but does not form well-defined Cu(II) or Zn(II) complexes under this modeling setup, and PpIX shows the excited-state and surface-localization profile expected of a photoactivatable, aggregate-associated species. Second, the wet-lab assays confirm the disease phenotype and show that the combined treatment is associated with significantly slower climbing decline (*p* = 2.39 × 10^–3^) and extended survival (log-rank *p* = 6.04 × 10^–13^) in Aβ42 flies. Third, the treatment is apparently tolerated in WT flies under descriptive comparison. What the data do not support is equally important: they do not identify which arm of the treatment drives the functional signal, do not demonstrate a treatment-mediated reduction of amyloid burden, do not establish protection against Cu(II)-mediated ROS, and do not constitute a formal test of additive or interactive effects between the two intervention components.

### 4.1 Major limitations of the present design

Before interpreting the computational and *in vivo* results, we state the experimental limitations that constrain what this manuscript can conclude. These are not opportunities for future work; they are structural boundaries on the present analysis. First, the study does not contain chelation-only or 5-ALA/light-only Aβ42 treatment arms. Without those arms, the observed climbing and survival differences between the untreated Aβ42 group and the Aβ42 + Treatment group cannot be attributed to the chelation component, the photodynamic component, or their combination, and no formal test of additive or interactive effects between the two components is possible. Second, treatment-group ThT amyloid burden was not measured. The ThT contrast reported in section “3.5 Wet-lab functional validation: partial climbing and lifespan rescue in Ab42-expressing flies” is therefore restricted to WT vs. untreated Aβ42 as disease-model validation and provides no evidence that the combined intervention reduces amyloid load. Third, direct PpIX and singlet-oxygen measurements were not performed, so the photodynamic mechanism of action of endogenously generated PpIX under 400 nm illumination in this model is not experimentally established. Fourth, biochemical readouts of ROS, brain metal levels, Aβ–metal interactions, and Aβ photooxidation were not performed; none of the individual steps of the proposed metal–Aβ–ROS mechanism has been directly measured in the treated animals. We confirm that these additional experiments are not available within the scope of the present revision and were not performed within the scope of this work. In consequence, every conclusion in the Results, Discussion, and Conclusion sections concerning the observed *in vivo* effect is kept strictly descriptive: the treated Aβ42 group climbed better and lived longer than the untreated Aβ42 group. The mechanistic content of the manuscript is confined to the computational predictions and is labeled as such throughout. Stated explicitly: the present study did not measure ROS, did not quantify PpIX, did not measure singlet oxygen, did not measure metal occupancy at Aβ, and did not perform any biochemical assay of the metal–Aβ–ROS axis in treated animals. The additional single-intervention control groups and biochemical measurements requested during peer review – including treatment-group ThT quantification, ROS measurements, PpIX quantification, and metal analyses – were not technically available for inclusion in the present revision. Consequently, the present manuscript remains an exploratory proof-of-concept study and does not experimentally establish the proposed molecular mechanism; the biological mechanism underlying the observed climbing and survival improvements remains undetermined.

### 4.2 An Fe-preferential rather than pan-metal chelation profile, and its limits

The chelation arm was designed around two competing requirements: strong enough coordination to plausibly influence redox-active metal chemistry, and mild enough behavior to leave essential metalloproteins largely intact. The CHELPG and Fe(III)-complex results are consistent with the first requirement inside the DFT model: deprotonated maltol localizes oxygen-donor electron density (−0.627, −0.641) at magnitudes comparable to deprotonated deferiprone (−0.636, −0.651), and the Fe(III)–maltol complex is converged and solvent-stabilized. The Cu(II) and Zn(II) results are the interpretively decisive ones. Whereas deferiprone forms Cu(II) and Zn(II) complexes with metal–oxygen distances of 1.94–1.97 Å (Cu–O) and 1.97–2.01 Å (Zn–O), and imidazole reference complexes show consistent Cu–N (∼2.01 Å), Zn–N (∼2.02 Å), and Fe–N (∼2.14–2.15 Å) distances, the optimized Cu(II)– and Zn(II)–maltol structures show elongated, incomplete Cu–O (2.824, 2.999 Å) and Zn–O (2.970, 2.125 Å) contacts. These distances lie outside typical coordinate-bond ranges. Rather than claiming broad multi-metal chelation, we interpret this as evidence that maltol supports Fe(III) coordination but does not, under this modeling setup, form well-defined Cu(II) or Zn(II) complexes.

The functional implication is directional but not clean. On the one hand, Fe is the metal most strongly implicated in Fenton-like ROS generation, and an Fe-preferential ligand is precisely what one would want if the goal were to weaken oxidative amplification at Aβ surfaces without indiscriminately disrupting Cu(II)-dependent enzymes (cytochrome c oxidase, Cu/Zn-SOD) or Zn(II)-dependent processes (synaptic Zn pools, the ∼300 known Zn metalloenzymes). On the other hand, Cu(II)–Aβ interactions are themselves a major contributor to amyloid-associated oxidative chemistry (Huang et al., 1999; Faller et al., 2014); a ligand that does not engage Cu(II) leaves that branch of the metal–Aβ–ROS loop intact. Two consequences follow. First, the present study explicitly does not demonstrate protection against Cu(II)-mediated ROS at Aβ; a distinct Cu(II)-coordinating ligand, or a maltol scaffold modified to broaden its metal-preference profile, would be required. Second, any behavioral or survival rescue observed *in vivo* cannot be attributed specifically to Fe-directed chemistry, because the Cu(II)–Aβ branch, the broad-spectrum 10 μM EDTA co-administered in the food, and the photodynamic arm are all present alongside maltol. The chelation contribution to the observed signal is therefore bounded above by “at most Fe-directed and EDTA-mediated” and cannot be finer without single-intervention arms.

### 4.3 PpIX as an aggregate-localizing, photoactivatable component

The PpIX side of the design is more directly supported. TD-DFT predicted excitations spanning the visible and near-UV, with the expected free-base porphyrin Q-band signature (519.6 and 496.9 nm; *f* = 0.0097 and 0.0033) and progressively stronger Soret-region and near-UV transitions (*f* = 0.140–0.353 between 391.0 and 343.6 nm). This is consistent with the expected photophysics of free-base porphyrins (Dougherty et al., 1998; Agostinis et al., 2011) and provides a mechanistic rationale for 400 nm illumination, which lies near the Soret region where the allowed transitions become substantial. TD-DFT does not, however, quantify singlet-oxygen yield, intersystem-crossing efficiency, or downstream ROS production; the claim is that PpIX is electronically photoactivatable, not that singlet-oxygen production has been confirmed in this system.

The docking result is qualitatively strong but interpretively conservative. PpIX engaged the Aβ42 fibril surface with lower Vina scores (−7.897 to −7.961 kcal/mol) than chlorin e6 (−6.952 to −6.978 kcal/mol) or maltol (−5.276 to −5.384 kcal/mol), and the top-ranked PpIX poses repeatedly converged on the central hydrophobic and turn-associated region (Leu17, Val18, Phe19, Phe20, Ala21, and downstream Asn27–Ile31) irrespective of which search box was used. Two interpretive points matter. First, Aβ42 fibrils do not present a classical binding pocket; Vina scores against a flat aggregate surface are best used as an ordinal comparison across ligands rather than as absolute affinities. Second, the residue-level convergence is the more informative finding: PpIX’s preferred contact surface is the hydrophobic/turn face of the fibril rather than the histidine cluster, consistent with a mechanism in which photoactivated PpIX would modify residues involved in aggregate-templated propagation rather than the residues that coordinate metals. Chlorin e6, a literature-validated Aβ photomodulator (Leshem et al., 2019), showed a similar contact geometry with weaker Vina scores; maltol’s contacts were uniformly weaker and less consistent, further supporting the assignment of maltol to the metal-coordination arm and PpIX to the aggregate-association arm.

### 4.4 What the functional and survival signals do and do not license

The *in vivo* results are directional but do not license mechanistic attribution to metal chelation, photodynamic action, or their interaction. Aβ42 + Treatment flies climb better than untreated Aβ42 flies across the 30-day time course (*p* = 2.39 × 10^–3^), still declining from a day-1 index of 0.769 to a day-30 index of 0.517 (a 32.8% decrease) but retaining measurable locomotor function to the end of the observation window. The survival curve is shifted substantially to the right (log-rank *p* = 6.04 × 10^–13^), with a median lifespan of approximately 40 d in the treated Aβ42 group. Untreated Aβ42 flies show the expected ThT elevation over WT (*p* = 1.99 × 10^–20^), validating the disease model. However, the treatment groups were not assayed for ThT, and the study therefore does not evaluate treatment-mediated reduction of amyloid burden. This is not a limitation that can be closed by reframing the existing data; it is a data gap that requires the ThT assay to be run in the Aβ42 + Treatment group. Until it is, the pre-specified multi-scale rescue index and amyloid–behavior–lifespan correlation analyses are not evaluable, and the behavioral and survival improvements can be discussed only as functional signals in the intact organism.

The alternative-explanation landscape is therefore wider than usual. The observed improvement could reflect (i) maltol-mediated Fe(III) sequestration weakening Fe-dependent oxidative chemistry at Aβ; (ii) EDTA-mediated general chelation of the fly gut/hemolymph metal pool creating a permissive chelation environment; (iii) endogenous PpIX accumulation followed by 400 nm-triggered aggregate-directed photooxygenation; (iv) any combination of (i)–(iii), including combinations that operate independently rather than synergistically; or (v) off-target effects of 5-ALA metabolism, EDTA at 10 μM, or repeated 400 nm exposure on non-Aβ processes. The design does not include the arms required to distinguish among these possibilities: chelation-only, 5-ALA/light-only, and if possible, individual maltol-only and 5-ALA/light-only conditions in the Aβ42 background, each with matched WT controls. Adding these arms is the most immediately productive next experiment.

Two additional data-boundary points bear on the interpretation. First, WT + Treatment climbing and survival trajectories overlap with untreated WT trajectories under descriptive comparison, and the WT + Treatment survival curve extends marginally beyond the WT curve at later time points, but a WT vs. WT + Treatment statistical contrast was not pre-specified. The tolerability observation is therefore descriptive; it is compatible with an absence of overt toxicity but does not license a claim of non-toxicity. Second, the 9 h : 15 h light : dark cycle used in this study is non-standard; all four groups are internally consistent because the schedule was constant across groups, but cross-study lifespan and behavioral comparisons should account for the difference relative to the more conventional 12:12 protocols in the *Drosophila* aging literature.

### 4.5 Level-of-theory and computational-model limitations

The chelation calculations were performed at B3LYP-D3BJ/def2-SVP for geometry optimization with def2-TZVP single points and CPCM/SMD water solvation. This level of theory is appropriate for qualitative geometric and electronic trends within a related ligand series, and is the standard first-pass choice for hydroxypyranone and hydroxypyridinone chelators; it is not, however, sufficient to obtain quantitative chelation-strength orderings for first-row transition metals. B3LYP systematically underestimates static correlation and can produce spin-state and ligand-field energy errors on the order of 5–15 kcal/mol for open-shell first-row transition-metal complexes, and def2-SVP is a modest basis set that can under-describe ligand polarization and metal–donor orbital overlap. Higher-level correlated methods – DLPNO-CCSD(T) with def2-TZVPP or larger basis sets, ideally combined with dielectric corrections at the same level of theory – would provide a more defensible quantitative ordering. Under the current level of theory, the appropriate reading of the maltol vs. deferiprone results is that both ligands are structurally compatible with Fe(III) coordination and that maltol does not form well-defined Cu(II) or Zn(II) complexes; magnitude claims about relative binding strength should not be extracted from ΔE_*bind*_ values computed here.

Two further computational-model constraints are worth naming. The competition calculations use imidazole as a minimal proxy for the Aβ N-terminal histidine cluster. Isolated imidazole reproduces the metal-coordinating side chain of a histidine but does not reproduce the Aβ backbone geometry, the N-terminal chelation with backbone amides that is characteristic of Cu(II)–Aβ, the flanking D1/E3/D7 acidic side chains that shape local electrostatics, or the aggregation-state effects that alter the effective coordination environment on fibril surfaces. A short N-terminal peptide such as DAEFRHDSGY (Aβ1–10) – or equivalent variants that retain the H6–D7 or H13–H14 pairs – would be the appropriate next-level substrate and is the natural computational follow-up. The TD-DFT analysis was performed on isolated PpIX rather than on a PpIX–Aβ complex; excited-state coupling to the peptide environment (which can shift both energies and oscillator strengths) is not modeled here. The docking analysis is subject to the pocket-less-target caveat already stated: Vina scores against Aβ42 fibrils are qualitative surface-localization indicators and should not be read as free energies of binding.

### 4.6 Scope: what would be required for mechanistic validation

The design that would be needed to convert the present computationally guided exploratory study into a mechanistically validated one is specified by the limitations enumerated in section “4.1 Major limitations of the present design.” Three additions are load-bearing. First, chelation-only (10 μM EDTA + 10 mM maltol) and 5-ALA/light-only Aβ42 arms – ideally accompanied by maltol-only and EDTA-only arms – each with matched WT controls, and with the WT vs. WT + Treatment tolerability contrast pre-specified. This is the design that would isolate the contribution of each intervention and permit a formal test of additive or interactive effects between the two intervention components. Second, ThT-derived amyloid burden measured in all four groups (WT, WT + Treatment, Aβ42, Aβ42 + Treatment) so the pre-specified multi-scale rescue framework and amyloid–behavior–lifespan correlations can be evaluated in their original form and any treatment effect on aggregate load can be tested directly. Third, at the computational side, the ΔE_competition calculations repeated with a short Aβ N-terminal peptide (e.g., DAEFRHDSGY) rather than imidazole, and the Cu(II)/Zn(II) complex calculations re-run at a higher level of theory (e.g., DLPNO-CCSD(T)/def2-TZVPP with CPCM/SMD) to determine whether the elongated Cu–O/Zn–O distances persist beyond B3LYP/def2-SVP. The computational-first framework generalizes: the same ground-state + excited-state + docking workflow can be reapplied to Cu(II)-coordinating ligands (to close the mechanistic gap identified in section “4.2 An Fe-preferential rather than pan-metal chelation profile, and its limits”), to BBB-tractable photosensitizers, or to alternative nodes of the metal–Aβ–ROS axis, and the resulting combined interventions can be pre-screened in tractable organismal systems before mammalian follow-up.

Three follow-up experiments would substantively strengthen the mechanistic interpretation of this specific design without altering the computational-first framing. (i) Add chelation-only (10 μM EDTA + 10 mM maltol) and 5-ALA/light-only Aβ42 arms – and if possible, maltol-only and EDTA-only arms – each with matched WT controls, and pre-specify the WT vs. WT + Treatment tolerability contrast. This would isolate the contribution of each intervention and permit a formal test of additive or interactive effects between the two intervention components. (ii) Assay ThT-derived amyloid burden in all four groups (WT, WT + Treatment, Aβ42, Aβ42 + Treatment) so the pre-specified multi-scale rescue index and amyloid–behavior–lifespan correlations can be evaluated in their original form. (iii) Repeat the ΔE_competition calculations with a short Aβ N-terminal peptide (e.g., DAEFRHDSGY) rather than imidazole, and re-run the Cu(II)/Zn(II) complex calculations for maltol at a higher level of theory (e.g., DLPNO-CCSD(T)/def2-TZVPP with CPCM/SMD) to determine whether the elongated Cu–O/Zn–O distances persist beyond B3LYP/def2-SVP. Together, these three experiments would let the study move from “consistent with” to “supports” for several of its central claims.

## 5 Conclusion

We designed a combined intervention against the Aβ42-associated metal–Aβ–ROS axis using a computational-first framework – combining ground-state DFT for maltol chelation, TD-DFT for PpIX excited-state photoactivation, and molecular docking for PpIX localization on the Aβ42 fibril surface – and then evaluated the same combined intervention as functional validation in a *Drosophila* pan-neuronal Aβ42 model as functional validation. The computational analyses in this manuscript partition the two ligands into complementary predicted niches: maltol shows features compatible with Fe(III) coordination but does not form well-defined Cu(II) or Zn(II) complexes under this modeling setup, and PpIX shows excited-state and surface-localization features consistent with photoactivatable, aggregate-associated behavior. Every claim in this paragraph is a computational prediction and none has been experimentally validated in this study. *In vivo*, the combined intervention is associated with slower climbing decline (*p* = 2.39 × 10^–3^), extended lifespan (log-rank *p* = 6.04 × 10^–13^), and overlapping WT and WT + Treatment trajectories under descriptive comparison. No attribution of the observed climbing or survival difference to metal chelation, photodynamic action, or their interaction is made; the additional experiments that would be required for such attribution – chelation-only and 5-ALA/light-only Aβ42 arms, treatment-group ThT amyloid burden, direct PpIX or singlet-oxygen quantification, and biochemical readouts of ROS, metal levels, Aβ–metal interactions, and Aβ photooxidation – were not available within the scope of the present revision and were not performed within the scope of this work.

Read strictly, the present manuscript reports a computationally guided exploratory proof-of-concept study. The study does not demonstrate treatment-mediated amyloid reduction, does not isolate the contribution of either arm of the combined intervention, does not constitute a formal test of additive or interactive effects between the two intervention components, and does not evidence protection against Cu(II)-mediated ROS. What it does demonstrate is that a computational-first design workflow – ground-state chelation chemistry, excited-state photophysics, and aggregate-surface docking – can jointly shape a computationally motivated combined intervention that is chemically coherent, showed overlapping WT and WT + Treatment trajectories under descriptive comparison, and was associated with a functional and survival signal in an intact organism model of Aβ42 toxicity. The utility of the framework lies in its transferability: the same workflow can be reapplied to Cu(II)-coordinating ligands, to BBB-tractable photosensitizers, and to alternative nodes of the metal–Aβ–ROS axis, and the resulting combined interventions can be pre-screened in tractable organismal systems before mammalian follow-up. The immediate priorities for this specific design are the missing single-intervention arms, treatment-group ThT quantification, and higher-level DFT calculations paired with a peptide-based mimic of the Aβ N-terminal metal-binding site. Accordingly, this study should be interpreted as a computationally guided exploratory investigation whose biological experiments establish only that the combined intervention was associated with improved behavioral and survival outcomes in this model. The mechanism underlying these improvements remains undetermined.

## Data Availability

The raw data supporting the conclusions of this article will be made available by the authors, without undue reservation.
